# Histone lactylation-ROS loop contributes to light exposure-exacerbated neutrophil recruitment in zebrafish

**DOI:** 10.1038/s42003-024-06543-5

**Published:** 2024-07-20

**Authors:** Cheng-zeng Qiu, Ren Zhou, Hao-yi Zhang, Ling Zhang, Zong-jun Yin, Da-long Ren

**Affiliations:** 1 College of Animal Science and Technology, Anhui Agricultural University, Hefei, 230036 China; 2 School of Life Science, Anhui Medical University, Hefei, 230032 China

**Keywords:** Neutrophils, Ageing

## Abstract

Light serves as a crucial external zeitgeber for maintaining and restoring physiological homeostasis in most organisms. Disrupting of light rhythms often leads to abnormal immune function, characterized by excessive inflammatory responses. However, the underlying regulatory mechanisms behind this phenomenon remain unclear. To address this concern, we use in vivo imaging to establish inflammation models in zebrafish, allowing us to investigate the effects and underlying mechanisms of light disruption on neutrophil recruitment. Our findings reveal that under sustained light conditions (LL), neutrophil recruitment in response to caudal fin injury and otic vesicle inflammation is significantly increased. This is accompanied by elevated levels of histone (H3K18) lactylation and reactive oxygen species (ROS) content. Through ChIP-sequencing and ChIP‒qPCR analysis, we discover that H3K18 lactylation regulates the transcriptional activation of the *duox* gene, leading to ROS production. In turn, ROS further promote H3K18 lactylation, forming a positive feedback loop. This loop, driven by H3K18 lactylation-ROS, ultimately results in the over recruitment of neutrophils to inflammatory sites in LL conditions. Collectively, our study provides evidence of a mutual loop between histone lactylation and ROS, exacerbating neutrophil recruitment in light disorder conditions, emphasizing the significance of maintaining a proper light-dark cycle to optimize immune function.

## Abstract (web summary)

Through ChIP-sequencing and ChIP‒qPCR analysis, authors discover a positive feedback loop between histone lactylation and reactive oxygen species which exacerbates neutrophil recruitment under light disorder conditions in Zebrafish.

## Introduction

Inflammation is a crucial signal that triggers the innate immune response and is a tightly regulated process involving the recruitment and activation of immune cells, including neutrophil^1,2^. Neutrophils are the most abundant immune cells in circulation and play a key role in the early stages of inflammation, acting as the first line of defense against pathogen invasion^3,4^. The recruitment of neutrophils to inflammatory sites is a complex process mediated by various molecular and cellular mechanisms necessary for tissue repair and pathogen elimination^2,5^.

Light, as a key zeitgeber, is crucial for maintaining and restoring circadian rhythms^6^, especially in zebrafish due to their in vitro development and optically transparent properties^7^. Studies have shown that a 5-6 light-dark (LD) cycle is required for establishing a robust rhythm in zebrafish embryos^8^. Previous studies have shown that light disruption is involved in regulating neutrophil migration in different models in mice and zebrafish^9,10^. However, the underlying mechanisms of light disturbance on the excessive recruitment of neutrophils remain unclear.

Lactylation is a newly discovered post-translational modification closely related to various biological processes, such as tumorigenesis, neurodevelopment, and inflammatory responses^11–13^. Lactic acid, a metabolite of glycolysis, significantly regulates the level of protein lactylation^14,15^. Histone lactylation has been shown to regulate inflammatory responses in macrophages, including cytokine secretion and cell activation^16^. However, the effects of histone lactylation on neutrophils have not been reported. Our study found that lactate content and histone lactylation levels changed in zebrafish under sustained light (LL) conditions, leading us to speculate that histone lactylation may participate in the regulation of exaggerated neutrophil recruitment under LL conditions in zebrafish.

Zebrafish have become an ideal model for studying light exposure and innate immune responses due to the optical transparency of their embryos and larvae^17^. Zebrafish are light-sensitive, and the dynamic migration of neutrophils during inflammatory responses can be monitored using a fluorescently labeled strain^1,18^. In this study, we will utilize in vivo imaging of zebrafish to explore the mechanisms by which constant light regulates neutrophil recruitment using tissue injury and LPS challenge models. We have concluded that a positive feedback loop of H3K18 lactylation-ROS contributes to the excessive recruitment of neutrophil caused by constant light exposure. Our study identify the mutual regulatory relationship between histone lactylation and ROS, providing a reasonable explanation for the abnormal recruitment of neutrophils under constant light exposure.

## Results

### Constant light increases the recruitment of neutrophil to inflammatory sites

To study the effects of constant light on neutrophil recruitment to tissue inflammation, we have constructed a light exposure model in zebrafish by continuously rearing embryos under constant light (LL) conditions for 4 days in our previous study^10^. The results showed that the migration of neutrophil to the inflammatory sites in zebrafish with light exposure was significantly increased^10^. However, the underlying regulatory mechanism of increased neutrophil migration remains unclear.

### Constant light exposure increases histone lactylation and ROS levels

**Fig. 1:**
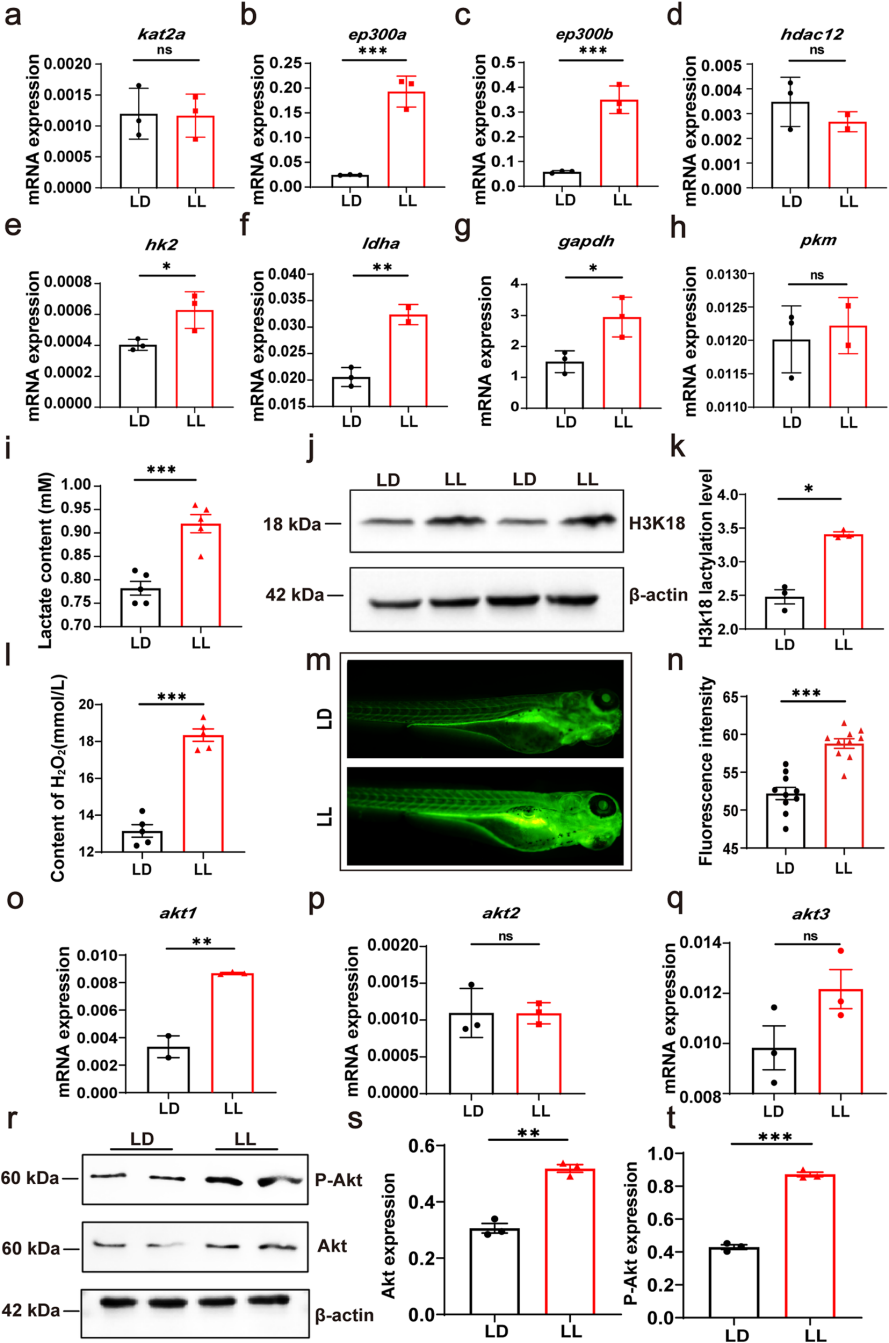
Sustained light increases H3K18 lactylation and ROS levels. **a**–**d** Q-PCR analysis showed that the expression levels of the lactylation-related genes *ep300a* and *ep300b* were significantly upregulated in juvenile fish reared under LL conditions. **e**–**h** Q-PCR analysis showed that the expression levels of the lactate metabolism-related genes *ldha* and *gapdh* were significantly upregulated in juvenile fish reared under LL conditions. **i** The lactate content in the juvenile fish reared under LL conditions was significantly increased. **j**, **k** Western blotting results showed that the level of histone lactylation (H3K18) was significantly upregulated in the juvenile fish of the LL group. **l** The hydrogen peroxide content in the juvenile fish under LL condition was significantly increased. **m**, **n** Fluorescent staining results showed that LL treatment significantly increased the levels of reactive oxygen species (ROS) in juvenile fish (*n* = 15). **o**–**q** Q-PCR analysis showed that the expression levels of *akt1* and *akt3* were upregulated under LL conditions, but *akt2 was not statistically significant*. Data were statistically analyzed using unpaired t tests. **r**–**t** Western blot analysis showed a significant increase in Akt phosphorylation levels in zebrafish under LL condition. All experiments were repeated three times. Bar graphs represent the mean ± standard error of the mean (SEM). (*p < 0.05; **p < 0.01; ***p < 0.001).

Lactylation has been reported to be involved in the regulation of inflammatory processes^16,19^. In this study, we hypothesized that under LL conditions, the lactate metabolism and lactylation levels in zebrafish would change and further affect neutrophil migration. We examined the expression of lactylation and lactate metabolism-related genes and found that under LL conditions, the *ep300a*, *ep300b*, *hk2*, *ldha*, and *gapdh* genes were significantly upregulated in zebrafish (Fig. 1a–h). Additionally, the lactate level in larvae significantly increased under LL condition (Fig. 1i), and western blot analysis indicated an increase in histone lactylation (H3K18) levels in zebrafish under LL condition (Fig. 1j, k). Furthermore, we found a significant increase in ROS level in zebrafish larvae under LL condition (Fig. 1l–n). In addition, we observed a significant increase in the expression of *akt1* and *akt3* under LL condition (Fig. 1o–q). Western blot analysis showed a significant increase in Akt phosphorylation levels in zebrafish under LL condition (Fig. 1r–t). These results indicate that zebrafish exhibit elevated lactate content, increased H3K18 lactylation level, and higher levels of ROS and Akt under LL condition.

### H3K18 lactylation regulates neutrophil recruitment

**Fig. 2:**
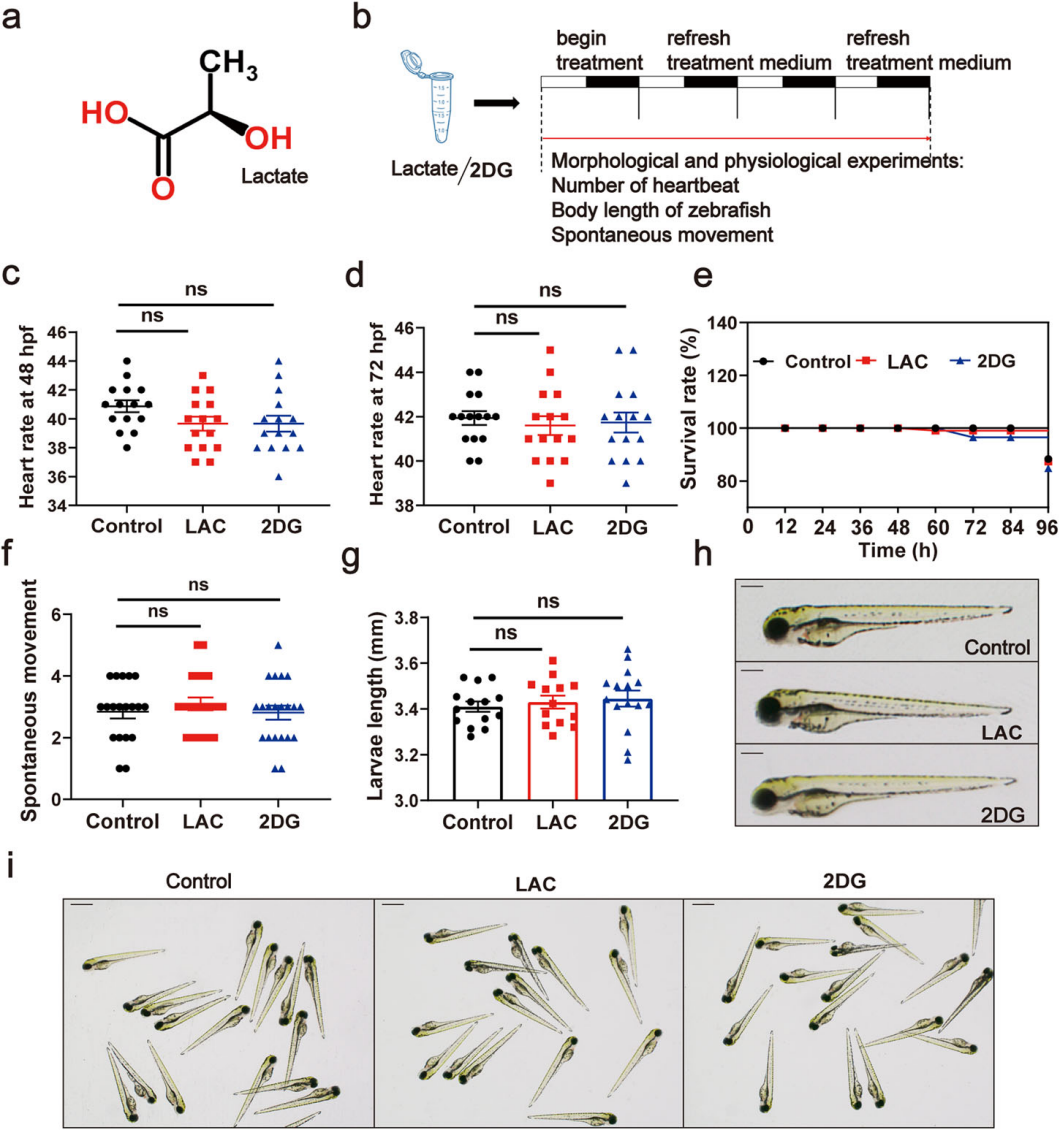
Evaluation of the physiological toxicity of lactic acid and 2DG on zebrafish larvae. **a** The chemical formula of lactic acid. **b** Experimental procedure for the effects of lactic acid and 2DG on zebrafish growth and development. **c** Heartbeat of 48 hpf zebrafish larvae (beats per minute, *n* = 15). **d** Heartbeat of 72 hpf zebrafish larvae (beats per minute, *n* = 15). **e** Survival rate curve of zebrafish larvae from 0 to 96 hpf (% survival, *n* = 15). **f** Tail swing frequency of 30 hpf zebrafish larvae (swing per minute, *n* = 15). **g** Statistical graph of body length of 96 hpf zebrafish larvae (mm, *n* = 15). **h**, **i** Morphological images of 96 hpf zebrafish larvae observed under a stereo microscope (scale bar: 350 μm). There were no significant differences in morphology between the lactic acid- and 2DG-treated groups compared to the control group. Data were analyzed using one-way ANOVA and log-rank test. All experiments were repeated three times. Bar graphs represent the mean ± standard error of the mean (SEM). (*p < 0.05; **p < 0.01; ***p < 0.001).

Thus, we intend to investigate whether lactylation of H3K18 regulates neutrophil recruitment under LL condition. We conducted experiments using exogenous lactic acid and 2-deoxyglucose (2DG) to manipulate the histone lactylation level in zebrafish under sustained light condition. To determine that the concentration of lactic acid and 2DG selected in following experiments had no significant toxicity of juvenile zebrafish, we designed the experimental procedure to observe heartbeat, survival rate, body length, and tail swing of larvae (Fig. 2a, b). The results showed that compared to the control group, exogenous lactic acid (100 mM) and 2DG (30 mM) had no significant influences on the heartbeat of zebrafish (48 hpf, 72 hpf) (Fig. 2c, d), survival rate from 0 to 96 hpf (Fig. 2e), tail swing at 30 hpf (Fig. 2f) and body length at 96 hpf (Fig. 2g). Furthermore, there were no significant differences in the overall morpha of zebrafish larvae among the three groups as observed by stereomicroscopy (Fig. 2h, i). These results indicated that the concentrations of lactic acid (100 mM) and 2DG (30 mM) used in the experiments did not have significant toxicity to the growth and development of zebrafish larvae.

**Fig. 3:**
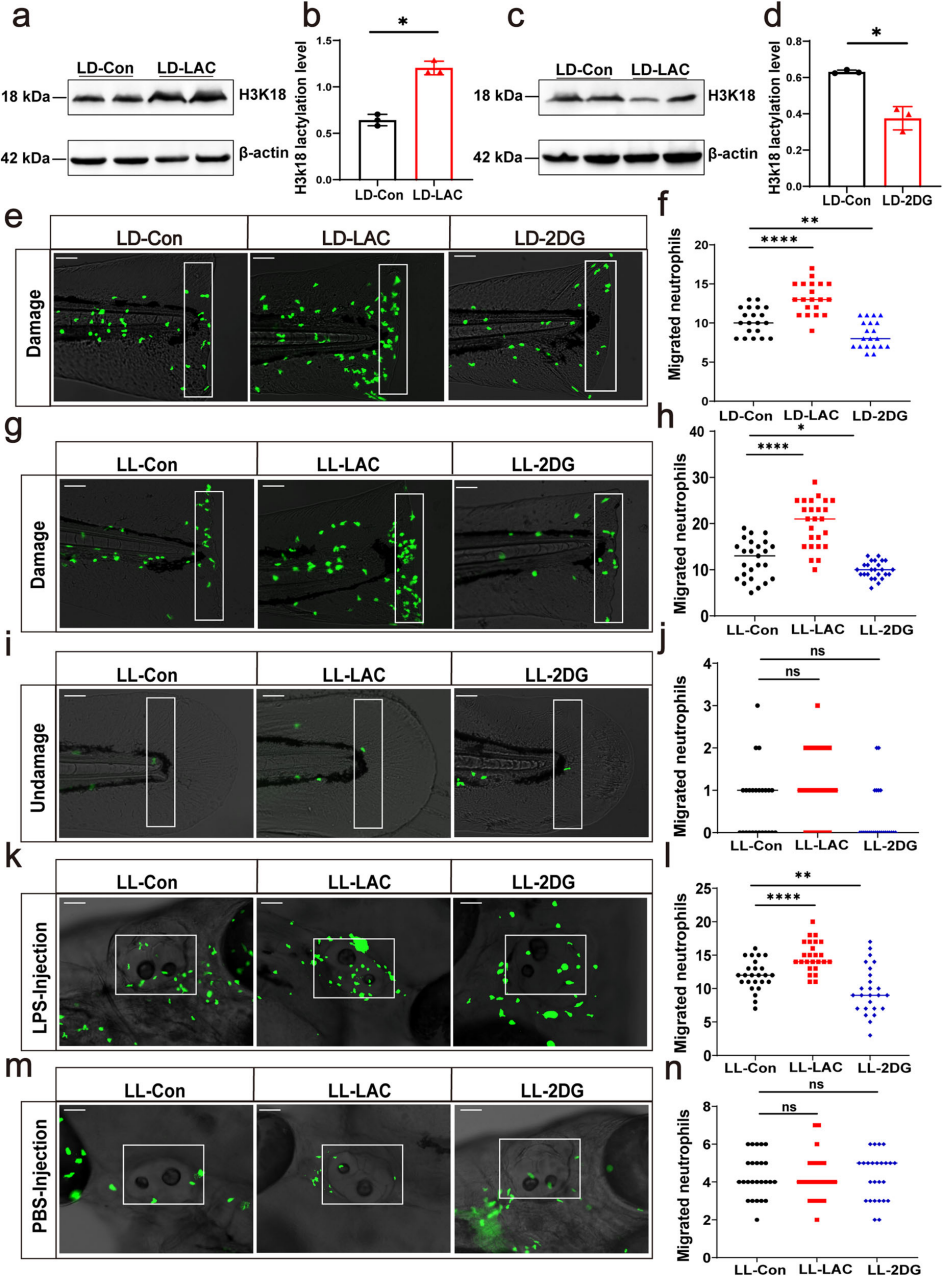
H3K18 lactylation regulates neutrophil recruitment. **a**, **b** Western blotting results showed that exogenous addition of lactic acid significantly enhanced the lactylation of H3K18. **c**, **d** Inhibition of lactate with 2DG resulted in a significant downregulation of H3K18 lactylation. **e**, **f** Statistical analysis showed that lactate significantly increased neutrophil recruitment at the site of injury under LD conditions, while 2DG significantly decreased neutrophil recruitment (*n* = 30). The white rectangles in the images represent the counting areas (scale bar: 200 μm). **g** Neutrophils migrated to the caudal fin injury site after 3 h of injury after lactic acid and 2DG treatment under LL condition. The white rectangles in the images represent the counting areas (scale bar: 200 μm). **h** Statistical analysis revealed that lactic acid significantly increased the recruitment of neutrophil to the injury site, while 2DG significantly decreased neutrophil recruitment (*n* = 30). **i**, **j** In larvae that were not subjected to fin injury, there was no significant difference in the neutrophil counts in the counting area between the control group and the treatment group. The white rectangles in the images represent the counting areas (scale bar: 200 μm) (*n* = 15). **k**, **l** Lactic acid increased the recruitment of neutrophils to otic vesicles 3 h after LPS challenge, while 2DG significantly decreased the recruitment (*n* = 30). The white rectangles in the images represent the counting areas (scale bar: 200 μm) (*n* = 30). **m**, **n** In larvae that were not subjected to microinjection of LPS, there was no significant difference in the neutrophil counts in the counting area between the control group and the treatment group. The white rectangles in the images represent the counting areas (scale bar: 200 μm) (*n* = 15). Unpaired tests and one-way ANOVA were used to analyze the differences. All experiments were repeated three times. Bar graphs represent the mean ± standard error of the mean (SEM). (*p < 0.05; **p < 0.01; ***p < 0.001).

Then, we observed that these concentrations of lactic acid and 2DG effectively upregulated and downregulated the level of H3K18 lactylation, respectively (Fig. 3a–d). Then, we found that under LD conditions, lactate and 2DG significantly increased and decreased the recruitment of neutrophils to the site of injury, respectively (Fig. 3e, f). Under LL conditions, the recruitment of neutrophils to injured caudal fin was also promoted by lactic acid and inhibited by 2DG at 3 h (Fig. 3g, h) and 6h (Supplementary Fig. 1a–d) after injury in the sustained light environment. Importantly, we observed that the increased neutrophil recruitment induced by lactate was due to enhanced inflammatory response rather than differences in the initial number of neutrophils in the tail fin (Fig. 3i, j). We validated these findings in the otic vesicle inflammatory model as well (Fig. 3k, l and Supplementary Fig. 2a–d). In the absence of LPS injection, there were no differences in neutrophil counts among the treatment and control groups (Fig. 3m, n). Overall, these results suggest that the manipulation of H3K18 lactylation level can regulate the recruitment of neutrophils in zebrafish.

### H3K18 lactylation regulates ROS levels and akt expression

**Fig. 4:**
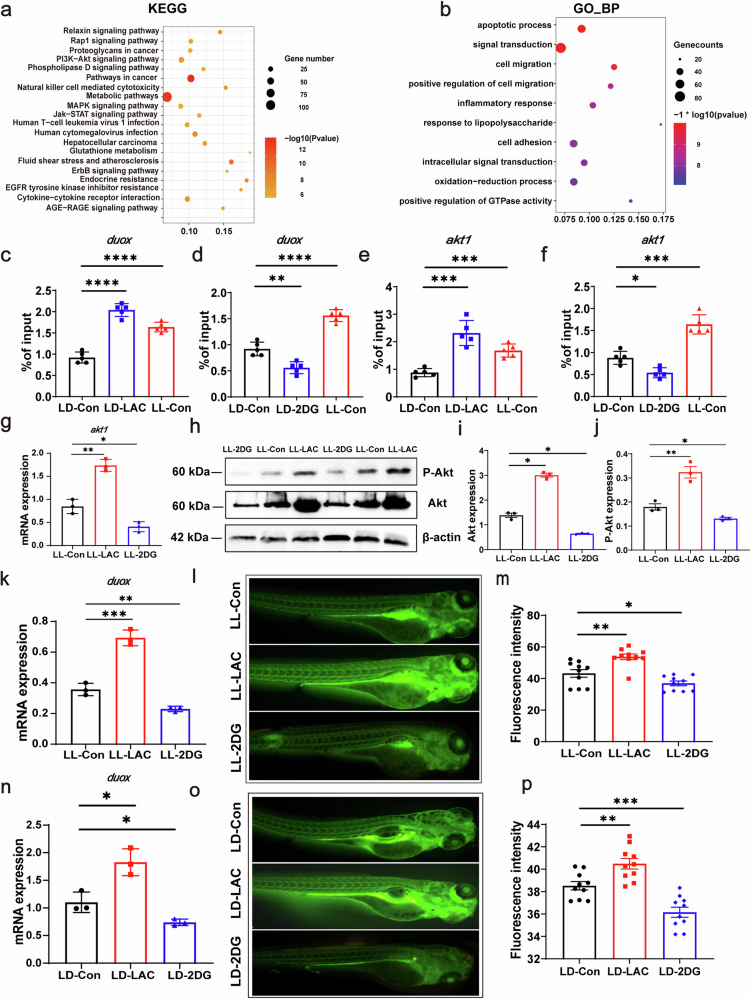
H3K18 lactylation regulates ROS levels and *akt* expression. **a** KEGG enrichment analysis showed the signaling pathways of genes bound by histone lactylation (H3K18). **b** GO-BP enrichment analysis showed the biological processes of genes bound by histone lactylation (H3K18). **c**–**f** ChIP-qPCR results showed that under LD conditions, lactylated H3K18 binds to the transcription region of *akt1* and *duox*, and similar results are observed under LL conditions. **g** Q-PCR showed that lactic acid significantly upregulated the expression of *akt1*, while 2DG significantly downregulated the expression of *akt1* under LL condition. **h**–**j** Western blotting showed that lactic acid and 2DG significantly upregulated and downregulated the expression of Akt protein under LL condition. **k** Q-PCR showed that lactic acid significantly upregulated the expression of *duox*, while 2DG significantly downregulated the expression of *duox* under LL condition. **l**, **m** Fluorescent staining results showed that lactic acid upregulated the levels of reactive oxygen species (ROS), while 2DG downregulated the levels of ROS under LL condition. **n** Q-PCR showed that lactic acid significantly upregulated the expression of *duox*, while 2DG significantly downregulated the expression of *duox* under LD condition. **o**, **p** Fluorescent staining results showed that lactic acid upregulated the levels of reactive oxygen species (ROS), while 2DG downregulated the levels of ROS under LD condition. Data were statistically analyzed using unpaired t tests and one-way ANOVA. All experiments were repeated three times. Bar graphs represent the mean ± standard error of the mean (SEM). (*p < 0.05; **p < 0.01; ***p < 0.001).

Continuous light exposure has been found to regulate the levels of ROS and Akt, which are crucial signals for neutrophil migration in zebrafish (Fig. 1). Based on this, we hypothesized that H3K18 lactylation may influence neutrophil migration by modulating the levels of ROS and Akt. To investigate this, we analyzed previously ChIP-sequence data from control and lactate-treated group in immune cell^11^ and found that lactylated H3K18 binds to promoter regions associated with ROS and Akt-related genes (Fig. 4a, b). We further confirmed this through ChIP-qPCR experiments, which showed that lactylated H3K18 binds to the transcription region of *duox* and *akt1* under LD conditions, and the binding was strengthen after lactate treatment and under LL conditions (Fig. 4c–f). Moreover, we observed that the expression and activation of Akt changed accordingly when the lactylation level of H3K18 was upregulated or downregulated by lactate and 2DG under LL conditions (Fig. 4g–j). Additionally, H3K18 lactylation significantly increased the expression of *duox* and the content of ROS under LL (Fig. 4k–m) and LD conditions (Fig. 4n–p) in zebrafish larvae. These findings suggest that H3K18 lactylation regulates the levels of ROS and Akt in zebrafish.

### ROS promote H3K18 lactylation and lactic acid metabolism

**Fig. 5:**
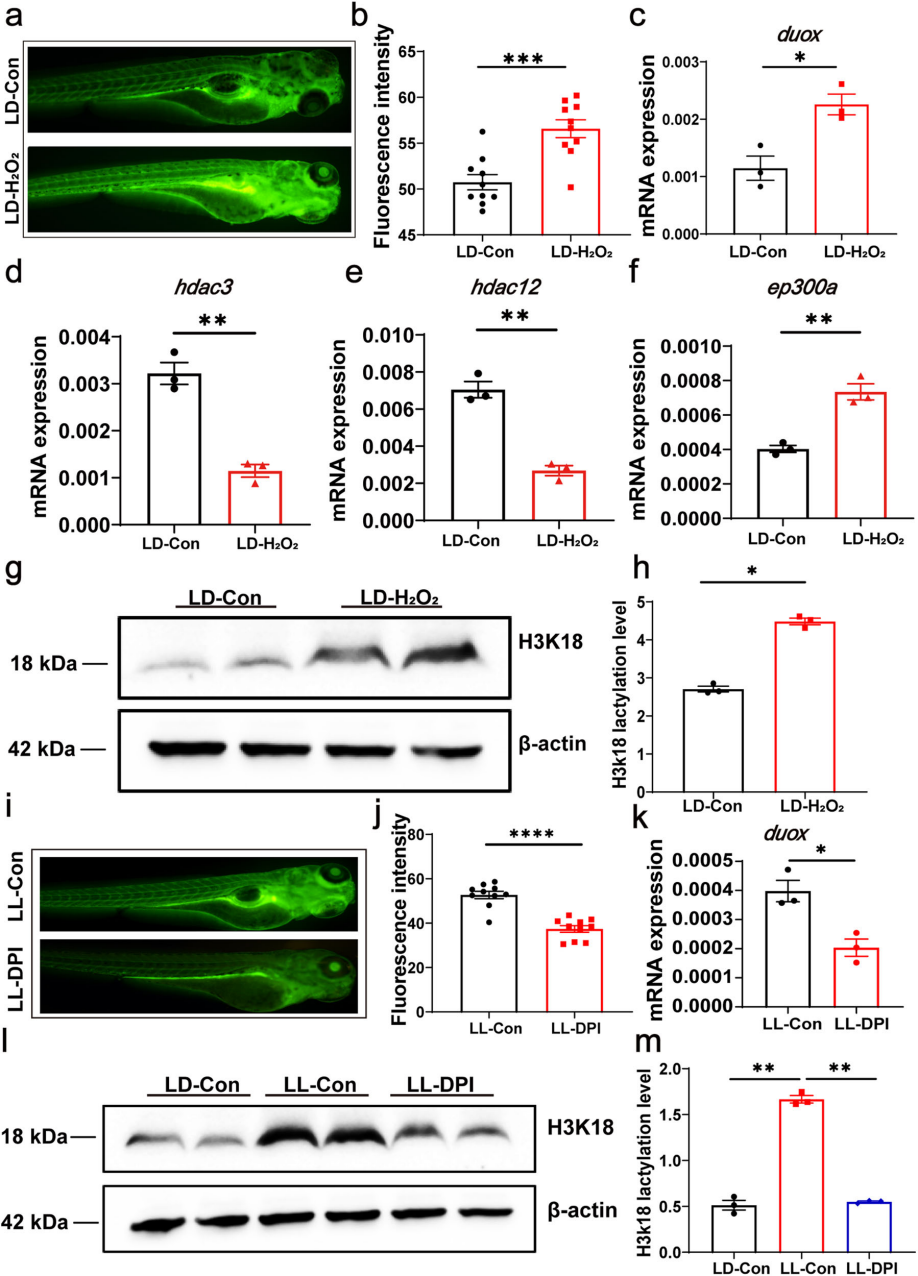
ROS promote H3K18 lactylation and lactic acid metabolism. **a**, **b** Fluorescence staining experiments revealed a significant increase in reactive oxygen species (ROS) levels in zebrafish larvae upon the addition of 100 μM hydrogen peroxide (*n* = 15). **c** Q-PCR results showed a significant upregulation of the oxidative stress gene *duox* upon treatment with 100 μM hydrogen peroxide. **d**, **e** The addition of 100 μM hydrogen peroxide significantly reduced the expression of the histone delactylation genes *hdac3* and *hdac12*. **f** The expression of the histone lactylation gene *ep300a* was significantly upregulated. **g**, **h** Treatment with 100 μM hydrogen peroxide significantly increased the level of histone lactylation (H3K18). **i**, **j** Fluorescence staining experiments showed a significant inhibition of reactive oxygen species (ROS) levels in zebrafish larvae under LL condition upon treatment with 10 μM DPI. **k** Treatment with 10 μM DPI significantly reduced the expression level of the oxidative stress gene *duox*. **l**, **m** DPI treatment significantly inhibited the H3K18 lactylation under LL condition. Data were statistically analyzed using unpaired t tests and one-way ANOVA. All experiments were repeated three times. Bar graphs represent the mean ± standard error of the mean (SEM). (*p < 0.05; **p < 0.01; ***p < 0.001).

Here, we aimed to further investigate whether ROS in turn modulate H3K18 lactylation. We used hydrogen peroxide to upregulate the ROS level and observed that it significantly increased the ROS intensity and *duox* expression in juvenile fish (Fig. 5a–c). Additionally, hydrogen peroxide treatment led to a reduction in the expression of two delactylation genes, hdac3 and hdac12 (Fig. 5d, e), while the expression of the lactylation gene ep300a was significantly increased (Fig. 5f). Western blotting results further confirmed that hydrogen peroxide significantly increased the H3K18 lactylation (Fig. 5g, h). To validate the regulatory effect of ROS on H3K18 lactylation, we treated zebrafish larvae with DPI to inhibit ROS production under LL condition. As a result, the ROS intensity and *duox* expression in juvenile fish were significantly downregulated after DPI treatment (Fig. 5i–k), and the addition of DPI also suppressed the level of H3K18 lactylation under LL condition (Fig. 5l, m). These findings provide strong evidence that reactive oxygen species can regulate the level of H3K18 lactylation.

### ROS and Akt regulate neutrophil recruitment under LL conditions

**Fig. 6:**
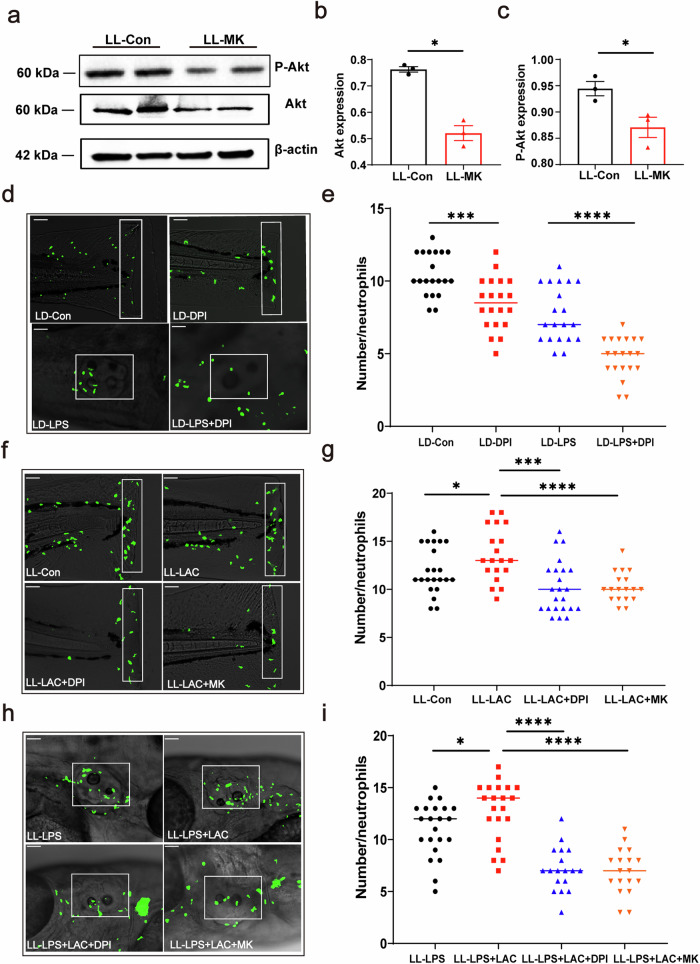
ROS and Akt regulate neutrophil recruitment under LL condition. **a**–**c** Western blotting results showed that MK-2206 significantly inhibited the expression of Akt. **d** The image showed that neutrophils migrated to the damaged caudal fin 3 h after injury after treatment with DPI. **e** The statistical graph shows that MK-2206 and DPI significantly inhibited the recruitment of neutrophils to the injured site of the caudal fin under LD condition (*n* = 20). **f** The image showed that neutrophils migrated to the damaged caudal fin 3 h after injury after treatment with MK-2206 and DPI. White rectangles represent counting areas (scale bar: 200 μm). **g** The statistical graph shows that MK-2206 and DPI significantly inhibited the recruitment of neutrophils to the injured site of the caudal fin under LL condition (*n* = 20). **h** The image showed that after treatment with MK-2206 and DPI, neutrophils migrated to the otic vesicle inflammation site 3 h after injury. White rectangles represent counting areas (scale: 200 μm). **i** The statistical graphs show that MK-2206 and DPI significantly inhibited the recruitment of neutrophils to the otic vesicle inflammation under LL condition (*n* = 20). The data were analyzed using unpaired t tests and one-way ANOVA. All experiments were repeated three times. Bar graphs represent the mean ± standard error of the mean (SEM). (*p < 0.05; **p < 0.01; ***p < 0.001).

We investigated the regulatory role of Akt and reactive oxygen species (ROS) in neutrophil recruitment in zebrafish under LL condition. We used MK-2206 and DPI to suppress Akt activation and ROS production. The results showed that MK-2206 treatment significantly downregulated the expression and activation of Akt protein (Fig. 6a–c). We confirmed that DPI significantly inhibited the recruitment of neutrophils to the site of injury/infection (Fig. 6d, e) under LD conditions. In both the caudal fin injury model (Fig. 6f, g) and the otic vesicle inflammatory model (Fig. 6h, i), both MK-2206 and DPI treatments significantly inhibited neutrophil recruitment and migration under LL condition. These findings suggest that the Akt pathway and ROS play a role in the regulation of neutrophil recruitment under LL condition.

## Discussion

The circadian rhythm is a 24-h cycle of physiological and behavioral changes that occur in organisms^20,21^. It plays a crucial role in regulating various physiological processes and behaviors, including temperature regulation, hormone secretion, and immune function^22–24^. The circadian rhythm is primarily regulated by endogenous molecular clocks, which are influenced by factors such as light intensity and environmental cycles^25–27^. This finding holds significant implications for comprehending the impact of light disruption on immune function and underscores the importance of maintaining an appropriate light-dark cycle to optimize immune responses.

Recent studies have shown a close association between light disruption and abnormal immune function^28,29^. However, the exact mechanisms underlying the regulation of light disruption in immune function are still unclear. To address this gap, our study utilized a zebrafish model to establish a light disruption model and confirmed the relationship between H3K18 lactylation, reactive oxygen species (ROS), and neutrophil recruitment under constant light conditions by the following points: Firstly, we found that under LL conditions, the level of H3K18 lactylation increases compared with LD conditions. By regulating the level of H3K18 lactylation, we found that H3K18 lactylation indeed regulates ROS levels. Through related ChIP-PCR experiments, we demonstrated that H3K18 binds to the key ROS regulating gene *duox* in zebrafish and found that lactylated H3K18 had a stronger binding ability to *duox* under LL condition compared with the LD condition. Conversely, by up-regulating and down-regulating ROS levels, the level of H3K18 lactylation undergoes corresponding changes. Together, These results demonstrated that there is a positive feedback loop between H3K18 lactylation and ROS in zebrafish.

Compared to previous studies that primarily used in vitro cell and mouse models, our use of zebrafish larvae offers several advantages. Zebrafish larvae have good optical transparency and are highly sensitive to the light environment^17^. Additionally, the dynamic migration behavior of neutrophils can be monitored in real time using transgenic labeled neutrophil strains^1,30^. Our findings provide a novel perspective on the regulatory mechanism of neutrophil recruitment under constant light exposure. We observed a significant increase in neutrophil recruitment in zebrafish following sustained light exposure. Furthermore, we discovered that this increased recruitment of neutrophils was aggravated by H3K18 lactylation-mediated modulation of ROS levels and the Akt pathway. While histone lactylation has been recently studied as a post-translational protein modification^31,32^, its role in neutrophils has been rarelyreported. Our study showed that manipulating H3K18 lactylation can significantly affect neutrophil recruitment in different inflammatory models in zebrafish. Interestingly, lactic acid, a major metabolite of anaerobic respiration, can increase lactylation levels, providing an additional explanation for the increased levels of inflammation under hypoxic conditions.

**Fig. 7:**
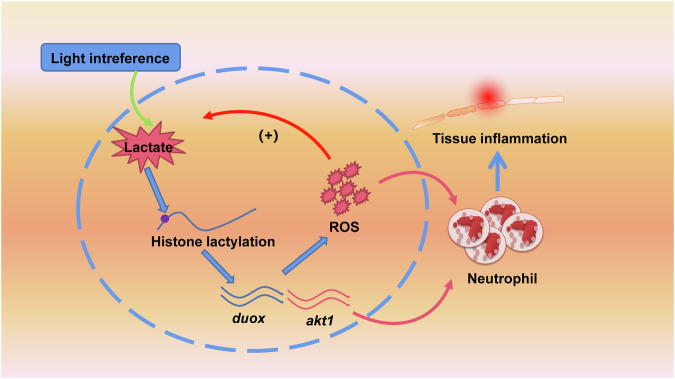
Illustration of the mechanism underlying the regulation of photoperiodic disruption in neutrophil recruitment. Photoperiodic interference affects the level of H3K18 lactylation, regulating the transcriptional activation of the *duox* gene and resulting in an increase in reactive oxygen species (ROS) levels. Additionally, H3K18 lactylation activates Akt expression. The generation of ROS further enhances the level of H3K18 lactylation, forming a positive feedback loop between histone lactylation and ROS. This positive feedback loop leads to excessive recruitment of neutrophil under LL condition.

Previous studies have suggested that H3K18 lactylation mainly promotes the transcriptional activation of related genes^33,34^. In line with these findings, we found that H3K18 lactylation can bind to the transcription region of the duox gene and promote its expression, leading to the production of more reactive oxygen species in zebrafish. This discovery highlights the mutual promotion of H3K18 lactylation and ROS expression, which explains the increased recruitment of neutrophils in the constant light environment. Our study provides evidence for this lactylation-ROS loop in zebrafish photo-induced neutrophil recruitment (Fig. 7), adding new insights to the field of histone lactylation and ROS.

While our study provides convincing evidence of the involvement of the lactylation-ROS loop in zebrafish photo-induced neutrophil recruitment. However, due to the lack of sufficient selectivity and specificity in the tools and drugs regulating lactylation, whether the lactylation modification of other proteins is involved in the regulation of neutrophil recruitment remains unknown and requires further exploration. Secondly, our study suggests that disruption of the photoperiod affected the level of ROS through lactylation modification; however, we cannot rule out the possibility that disruption of the photoperiod directly affects the level of ROS. Previous studies have shown that changes in the light-dark cycle can directly affect the generation and clearance mechanisms of ROS^35^. Therefore, it is possible to speculate that disruption of the photoperiod directly affects the level of ROS, thus affecting neutrophil migration. Currently, the Akt pathway is widely recognized as a signaling pathway that influences the migration of neutrophils^36,37^. Although the study demonstrates that histone lactylation regulated the recruitment of neutrophils through the ROS/Akt pathway in animal level. However, it remains unclear whether this regulation of neutrophils in a cell-autonomous manner. Further approaches, such as conditional knockout of these genes in neutrophils in zebrafish, were needed to explore the phenomenon in future study.

In conclusion, our study emphasizes the role of the lactylation-ROS loop in exacerbating zebrafish photo-induced neutrophil recruitment. These findings not only contribute to the study of photodisturbance but also offer potential research directions in other inflammation-related pathological processes associated with lactylation modifications or reactive oxygen species. The identification of this feedback loop provides valuable insights into the interplay between metabolic processes and immune cell dynamics.

## Methods

### Zebrafish strains and maintenance

Zebrafish embryos were obtained by natural mating of wild-type (WT/AB) and transgenic line Tg(*lyz*:EGFP) at 28.5 °C. The Tg(*lyz*:EGFP) line labels neutrophil by expressing the fluorescent protein EGFP^38^. The obtained embryos were reared in petri dishes with Hanks medium (0.8 g/L NaCl, 0.04 g/L KCl, 0.385 mg/L Na_2_HPO_4_, 0.6 mg/L KH_2_PO_4_, 0.144 g/L CaCl_2_, 0.246 g/L MgSO_4_ and 0.35 g/L NaHCO_3_) containing 1 mg/L Methylene Blue. Wild-type (WT/AB) and transgenic line Tg(*lyz*:EGFP) zebrafish embryos were raised at a constant temperature of 28.5 °C, pH 6.5-7.5, 14 h/10 h light-dark (LD), and constant light (LL) conditions, respectively. After naturally mating and collecting eggs from 300 zebrafish, the embryos were placed in petri dishes containing Hanks solution, and then incubated in a light incubator under constant illumination for 4 days to establish a zebrafish light interference model. Simultaneously, 300 zebrafish embryos were collected and subjected to normal light-dark conditions (14 h of light, 10 h of darkness) to serve as the control group. The stage of embryos and larvae were defined as hours post fertilization (hpf) or days post fertilization (dpf). We strictly adhered to the guidelines and regulations set forth by the Animal Resource Centre and the Animal Care and Use Committee of Anhui Agricultural University (SYXK (Anhui) 2016-007), and all items conformed to the guidelines of the China Academy of Food and Drug Inspection and Quarantine on the care and use of laboratory animals.

### Behavioral monitoring

For behavioral analysis, zebrafish larvae raised in normal light-dark (LD) and constant light (LL) conditions (0–96 hpf) were tested by placing them in 48-well plates at 5 dpf, with 24 larvae in each group. Experiments were conducted under constant darkness (DD) condition and the test procedure was set up as follows: continuous darkness for 3 days (infrared illumination was used continuously throughout) for 3 days, and constant temperature conditions of 28.5 °C were maintained. Movement of juveniles was detected for 4 consecutive days using a behavioral recording analysis system (ViewPoint, France) and the total distance moved by one juvenile in 10 min was recorded using Zebralab 3.11 software (ViewPoint, France).

### Caudal fin damage and imaging

Tg (*lyz*:EGFP) larvae reared under different conditions were anaesthetized using 0.1 g/mL MS-222 solution (Sigma, E10521) when juveniles were cultured to 5 dpf. Caudal fin wounding was performed on juveniles at the end of the spinal cord using a sterile scalpel blade under a microscope and embryo medium was used to recover the injured juveniles. The migration of caudal fin neutrophils was observed under a fluorescence microscope at 3 h after injury, and the number of neutrophil was analyzed using ImageJ software.

### Otic vesicle inflammation model

N-phenylthiourea (PTU, Sigma) was used to prevent pigmentation in juvenile fish. Tg(*lyz*:EGFP) larvae cultured under LD and LL conditions were anaesthetized with 0.1 g/mL MS-222 solution (Sigma, E10521). LPS (0.2 mg/ml, Sigma, L5293) was dissolved in PBS and injected into the otic vesicles of juvenile fish. Equal doses of PBS injections served as control group. Three hours after LPS injection, neutrophil migration was observed under a fluorescence microscope, and the number of neutrophils migrating to the otic vesicles was analyzed by ImageJ software.

### RNA extraction and RT‒qPCR

Using Trizol reagent (Takara, Japan), total RNA from juvenile fish under different culture conditions was extracted, and the RNA was reverse transcribed into cDNA (Vazyme, China) and diluted 5-fold in dd H_2_O (double distilled) followed by qPCR. Quantitative real-time fluorescence qPCR (qPCR) was performed in a LightCycler® 96 instrument (Roche) using SYBR Premix® Ex Taq™ (Takara, RR430S). At least three independent samples were tested per experiment. Relative expression levels were normalized to β-actin using the 2^−ΔΔCT^ method.

### Drug treatments

Lactic acid (100 mM) (MACKLIN, China) and 2-deoxyglucose (2DG) (30 mM) (MACKLIN, China) were selected to treat 5 dpf zebrafish juveniles by soaking for 3 h to regulate the lactylation level and evaluate the physiological function of larvae. DPI (10 µM) (HY-100965, MCE) was used to treat zebrafish larvae at 4 dpf for 3 h to inhibit ROS production. The larvae were then stained to detect reactive oxygen species (ROS) levels using a DC-FHDA fluorescent probe (MCE, USA). Zebrafish juveniles were treated with H_2_O_2_ (Solarbio, China) for 3 h to detect H_2_O_2_ content under different light conditions. MK-2206 (2 μM) (Selleckchem, USA) was used to inhibit Akt activation in larvae.

### Physiological assessment of zebrafish larvae

To evaluate the toxicity of lactic acid (100 mM) and 2-DG (30 mM) on the growth and development of zebrafish larvae, zebrafish larvae at 5 dpf were immersed in lactic acid (100 mM) and 2-DG (30 mM) for 3 h to assess their heart rate, spontaneous movement, survival rate, and changes in body length and morphology. Specifically, the experiment included a control group, a lactic acid (100 mM) treatment group, and a 2-DG (30 mM) treatment group, with each group consisting of three parallel sets, each containing 15-20 zebrafish embryos. The drug was refreshed every 24 h. Subsequently, a series of physiological and developmental indicators such as spontaneous movement, heart rate, morphology, body length, and survival rate will be observed in zebrafish from 0 hpf to 96 hpf.

### Western blotting

To analyze the level of histone lactylation under different light conditions, protein samples were collected from 50 juvenile fish under LD (normal light) and LL (continuous light) conditions, lysed and subjected to Western blotting using RIPA lysis buffer (Servicebio, China). The collected protein samples were boiled for 5 min, separated using a 12% SDS‒PAGE gel, transferred to a nitrocellulose membrane (Servicebio, China), soaked in 5% skimmed milk for 2 h, and incubated overnight at 4 °C with primary antibodies against Akt (CST, 4691T) and H3K18la (PTMBIO, PTM-1427RM). After washing, the HRP-labeled secondary antibody (Sangon, D111042-0100) was incubated for 2 h at room temperature and photographed with chemiluminescent solution. The protein expression was calculated from the ratio of the gray value of the target protein to the housekeeping protein. The gray values of each set of protein bands were calculated by ImageJ. Significance analysis was performed by GraphPad Prism 8.4.3.

### Lactate content assessment

The lactate content of zebrafish juveniles under different light conditions was determined using the Lactate Assay Kit (Abbkine, USA). Briefly, 40 juvenile samples were collected under LD and LL conditions, and the samples were added to the extract solution, mixed well, and then centrifuged at 12,000 × *g* for 10 min. The supernatants were placed in a chemiluminescence detector to determine the absorbance value.

### H_2_O_2_ content assessment

The H_2_O_2_ content of zebrafish larvae under different light conditions was determined using an H_2_O_2_ Content Assay Kit (Solarbio, China). Forty zebrafish larvae kept under LD and LL conditions were collected, and 500 μL of pre-cooled acetone solution at 4 °C was added to each, and centrifuged with 8000 r/min at 4 °C for 10 min. The supernatants were placed in a chemiluminescence detector to determine the absorbance value.

### Chromatin immunoprecipitation (ChIP) assays

ChIP experiments were conducted according to the manufacturer’s protocol (Millipore, USA). Briefly, approximately 50 larvae were collected and cross-linked in 2% formaldehyde at room temperature for 35 min. and then cross-linking was stopped by adding 1/10 V of 1.25 M glycine, followed by PBS washing (3 times, each for 10 min). The following procedures were performed according to the manufacturer’s protocol (Millipore, USA). The specific antibody of H3K18la (PTMBIO, PTM-1427RM) was used to precipitate protein-DNA complexes. Rabbit resourced IgG (Invitrogen, USA) was used as a negative control. ChIP‒qPCR was performed using primers flanking the initiation site in the promoter.

### ChIP-sequencing analysis

To study the function of different binding genes of H3K18 in immune cells, we conducted functional enrichment analysis, including Kyoto Encyclopedia of Genes and Genomes (KEGG) and Gene Ontology (GO) analysis from published Chip-seq data^11^. Firstly, differential analysis was performed using edgeR, selecting significantly differentially expressed genes with a P-value less than 0.05 after FDR correction. Subsequently, samples were annotated using an online annotation tool. Next, differential gene IDs were converted to ENTRE IDs using R software, with a set threshold of 0.05 for both P-value and Q-value, for KEGG and GO enrichment analysis. We conducted gene ID transformation analysis on the online website DAVID (DAVID: Functional Annotation Tools (ncifcrf.gov)) to investigate the biological processes of these genes. Subsequently, in R (4.2.1), we visualized the enrichment analysis using the ggplot2 (3.3.6) R package.

### Physiological and morphological assessment

Swaying rate at 30 hpf (time/1 min), heart rate at 48 hpf and 72 hpf (time/20 s), body length at 96 hpf (cm), and survival rate at 96 hpf after lactic acid (100 mM) and 2DG (30 mM) immersion treatments were observed by a stereomicroscope. Each group of approximately 30 zebrafish was used in the experiment^39^.

### ROS staining

To measure the levels of reactive oxygen species (ROS) in zebrafish larvae, zebrafish larvae were treated with the DC-FHDA fluorescent probe (1 μM) (MCE, USA), and incubated at 37 °C in a constant-temperature incubator for 1 h. Subsequently, images were captured under a fluorescence microscope, and the fluorescence intensity of the larvae was quantified using ImageJ software.

### Statistics and reproducibility

At least three biological samples were used for each experiment. All experiments were independently repeated three times. GraphPad Prism 8.4.3 was used to analyze the experimental data. Data were analyzed using unpaired t tests and one-way ANOVA and are displayed as the mean ± SD. Differences between means were considered significant at *P < 0.05, **P < 0.01, ***P < 0.001, ****P < *0.0001*.

### Reporting summary

Further information on research design is available in the Nature Portfolio Reporting Summary linked to this article.

### Supplementary information

supplementary information

Description of Additional Supplementary Files

Supplementary data1

Reporting Summary

## Data Availability

All data generated during our study are fully documented in the published article and its supplementary information. The source data behind the graphs in the paper are provided in Supplementary Data 1.
