# Supplementary material for: Histone lactylation-ROS loop contributes to light exposure-exacerbated neutrophil recruitment in zebrafish
Source: Commun Biol. 2024 Jul 20;7:887. doi: 10.1038/s42003-024-06543-5 (PMC11271584; doi:10.1038/s42003-024-06543-5)
Supplement: Supplementary file 1 — supplementary information [file 42003_2024_6543_MOESM1_ESM.pdf]

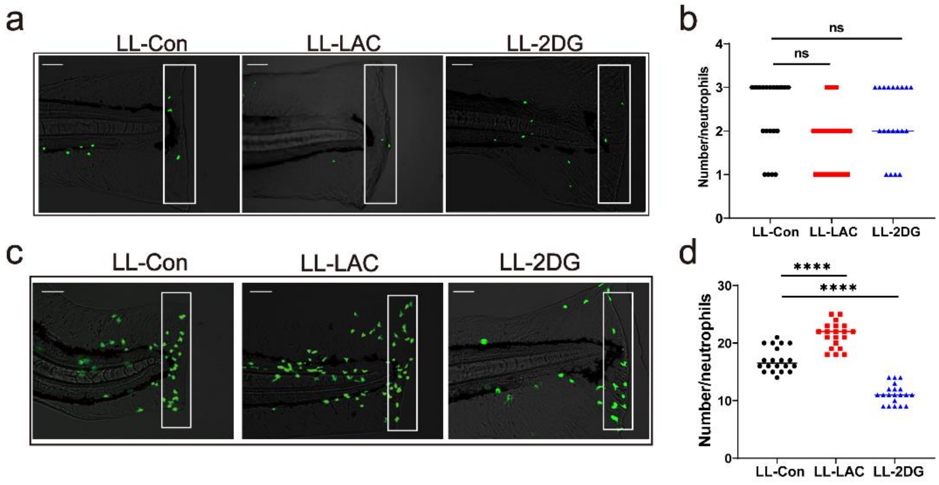


**Supplementary Figure 1 Neutrophil recruitment at the site of injury at 0 h /6 h after injury.** (a, b) Fluorescent images showed migrating neutrophils. White rectangles indicate the counting area (scale bar: 200 μm). Statistical analysis showed no significant difference in the number of neutrophils at the site of injury at 0 h (n=20). (c, d) Statistical analysis revealed that lactic acid significantly increased the recruitment of neutrophil to the injury site, while 2DG significantly decreased neutrophil recruitment (n=20). Bar graphs represent the mean ± standard error of the mean (SEM).

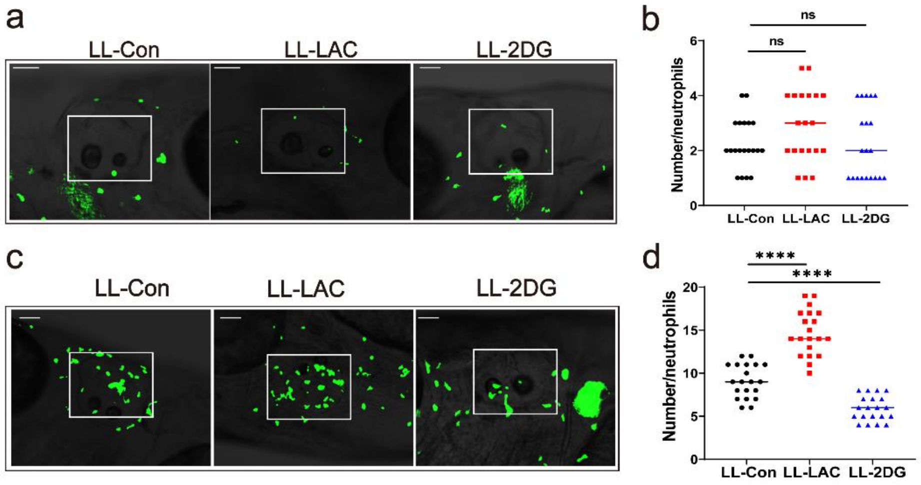


**Supplementary Figure 2 Neutrophil recruitment at 0 h/6 h after LPS injection following lactic acid and 2DG treatment.** (a, b) Fluorescent images showed migrating neutrophils. White rectangles indicate the counting area (scale bar: 200 μm). Statistical analysis showed no significant difference in the number of neutrophils at the site of injury at 0 h (n=20). (c, d) Statistical analysis revealed that lactic acid significantly increased the recruitment of neutrophil to the injury site, while 2DG significantly decreased neutrophil recruitment (n=20). Bar graphs represent the mean ± standard error of the mean (SEM).
