# Supplementary material for: Histone lactylation-ROS loop contributes to light exposure-exacerbated neutrophil recruitment in zebrafish
Source: Commun Biol. 2024 Jul 20;7:887. doi: 10.1038/s42003-024-06543-5 (PMC11271584; doi:10.1038/s42003-024-06543-5)
Supplement: Supplementary file 3 — Supplementary data1 [file 42003_2024_6543_MOESM3_ESM.pdf]

Figure1A

	LD			LL	
0.00108	0.00086	0.00165	0.00079	0.00147	0.00124

Figure1B

	LD			LL	
0.02435	0.02385	0.02538	0.15713	0.21316	0.20877

Figure1C

	LD			LL	
0.06037	0.06164	0.05256	0.33448	0.4118	0.30355

Figure1D

	LD			LL	
0.00383	0.00425	0.00236		0.00296	0.00239

Figure1E

	LD			LL	
0.00037	0.00044	0.0004	0.00067	0.0005	0.00072

Figure1F

	LD			LL	
0.02047	0.01884	0.02241		0.03373	0.03103

Figure1G

	LD			LL	
1.60214	1.11729	1.8025	2.96905	3.5801	2.2974

Figure1H

	LD			LL	
0.01144	0.01226	0.01234	0.01252		0.01192

Figure1I

LD	LL
0.75	0.85
0.82	0.95
0.78	0.91
0.81	0.93
0.75	0.96

Figure1K

LD	LL
2.27369	3.47906
2.62521	3.38059
2.53648	3.36152

Figure1L

LD	LL
12.53	18.45
13.51	18.78
14.23	19.32
12.35	17.54
13.12	17.65

Figure1N

LD	LL
65.32	75
70.21	78
68.32	74
72.15	81
55	83
59	79
62	69
65	78
64	79
63	80

Figure1O

LD			LL	
0.00413	0.00254	0.00867	0.00879	0.00861

Figure1P

	LD			LL	
0.00088	0.00148	0.00093	0.00094	0.00111	0.00123

Figure1Q

	LD			LL	
0.00962	0.01144	0.00843	0.0137	0.01168	0.01113

Figure1S

LD	LL
0.29472	0.52691
0.33942	0.53717
0.28423	0.4913

Figure1T

LD	LL
0.40298	0.86048
0.43321	0.89966
0.45231	0.85493

Figure2C

Control	LAC	2DG
40	41	38
39	38	44
42	39	40
44	37	38
41	40	40
41	40	39
40	37	43
42	39	39
41	40	38
39	38	38
42	38	39
41	41	42
40	43	41
38	42	36
43	42	40

Figure2D

Control	LAC	2DG
41	40	42
42	41	39
43	42	40
42	42	42
44	41	43
42	40	41
	43	42
43	44	45
42	41	41
44	40	40
41	42	45
41	45	43
40	43	42
42	39	41
40	41	40

Figure2E

Group	Time（h）	control														
Control	0	0	0	0	0	0	0	0	0	0	0	0	0	0	0	0
	12	0	0	0	0	0	0	0	0	0	0	0	0	0	0	0
	24	0	0	0	0	0	0	0	0	0	0	0	0	0	0	0
	36	0	0	0	0	0	0	0	0	0	0	0	0	0	0	0
	48	0	0	0	0	0	0	0	0	0	0	0	0	0	0	0
	60	0	0	0	0	0	0	0	0	0	0	0	0	0	0	0
	72	0	0	0	0	0	0	0	0	0	0	0	0	0	0	0
	84	0	0	0	0	0	0	0	0	0	0	0	0	0	0	0
	96	0	0	0	0	0	0	0	0	0	0	0	0	0	0	0

Group	Time（h）	LAC														
Control	0	0	0	0	0	0	0	0	0	0	0	0	0	0	0	0
	12	0	0	0	0	0	0	0	0	0	0	0	0	0	0	0
	24	0	0	0	0	0	0	0	0	0	0	0	0	0	0	0
	36	0	0	0	0	0	0	0	0	0	0	0	0	0	0	0
	48	0	0	0	0	0	0	0	0	0	0	0	0	0	0	0
	60	0	0	0	0	0	0	0	0	0	0	0	0	0	0	0
	72	0	0	0	0	0	0	0	0	0	0	0	0	0		
	84	0	0	0	0	0	0	0	0	0	0	0	0	0		
	96	0	0	0	0	0	0	0	0	0	0	0	0	0		
LAC	0	0	0	0	0	0	0	0	0	0	0	0	0	0	0	0
	12	0	0	0	0	0	0	0	0	0	0	0	0	0	0	0
	24	0	0	0	0	0	0	0	0	0	0	0	0	0	0	0
	36	0	0	0	0	0	0	0	0	0	0	0	0	0	0	0
	48	0	0	0	0	0	0	0	0	0	0	0	0	0	0	0
	60	0	0	0	0	0	0	0	0	0	0	0	0	0	1	0
	72	0	0	0	0	0	0	0	0	0	0	0	0	0		
	84	0	0	0	0	0	0	0	0	0	0	0	0	0		
	96	0	0	0	0	0	0	0	0	0	0	0	0	0		

Group	Time（h）	2DG														
2DG	0	0	0	0	0	0	0	0	0	0	0	0	0	0	0	0
	12	0	0	0	0	0	0	0	0	0	0	0	0	0	0	0
	24	0	0	0	0	0	0	0	0	0	0	0	0	0	0	0
	36	0	0	0	0	0	0	0	0	0	0	0	0	0	0	0
	48	0	0	0	0	0	0	0	0	0	0	0	0	0	0	0
	60	0	0	0	0	0	0	0	0	0	0	0	0	0	0	0
	72	0	0	0	0	0	0	0	0	0	0	0	0	0		1
	84	0	0	0	0	0	0	0	0							
	96	0	0	0	0	0	0	0	0							

Figure2F

Control	LAC	2DG
2	3	1
2	3	4
3	4	2
4	2	3
3	4	3
2	2	1
1	3	3
3	3	4
4	2	3
2	3	3
3	5	2
3	2	5
3	3	3
1	4	2
3	5	4
3	4	3
4	2	4
4	3	2
4	3	2
	2	3
	3	2

Figure2G

Control	LAC	2DG
3.52512	3.37922	3.5157
3.37097	3.34004	3.6608
3.53678	3.36701	3.4149
3.31352	3.49053	3.5606
3.2785	3.55006	3.6269
3.53676	3.50567	3.4413
3.39791	3.50126	3.5013
3.35698	3.32017	3.5
3.43121	3.28208	3.2107
3.44755	3.61065	3.2993
3.3484	3.32747	3.5124
3.45286	3.42332	3.4233
3.43045	3.48611	3.4113
3.30943		3.1774
		3.419

Figure3B

LD	LD+LAC
0.58108	1.1636
0.70455	1.28863
0.64135	1.15949

Figure3D

LD	LD+2DG
0.64214	0.30306
0.62767	0.39745
0.62354	0.42536

Figure3F

11	13	8
12	11	6
8	12	7
9	15	9
10	14	10
12	13	10
13	16	11
9	11	8
10	13	7
11	13	9
10	11	8
8	15	11
8	9	7
11	15	11
10	12	7
13	15	6
12	13	10
8	11	11
12	17	7
8	15	7

Figure3H

18	17	8
17	12	9
16	21	12
8	12	7
7	21	12
14	23	9
15	25	13
11	29	8
14	18	11
15	23	13
8	15	10
11	15	9
16	19	9
15	15	8
9	25	10
10	23	11
19	25	10
18	25	6
11	10	12
13	15	10
7	25	12
6	26	9
8	23	10
14	21	9
5	17	11

Figure3J

0	0	0
0	0	0
0	0	0
0	0	0
0	0	0
0	0	2
1	0	2
1	1	0
1	1	0
1	1	0
1	1	0
1	1	0
1	2	0
1	2	0
1	2	0
3	2	0
1	3	0
0	2	0
2	1	0
2	1	0
0	1	0
0	1	1
0	1	1
0	2	1
0	2	1

Figure3L

9	14	3
12	11	5
11	15	10
15	14	9
12	18	9
11	15	8
10	12	16
12	14	6
13	15	7
8	20	14
16	14	13
10	16	15
11	14	9
7	13	14
11	17	12
15	14	10
13	17	9
13	18	7
14	17	10
15	17	7
12	15	6
11	14	17
12	13	7
15	12	11
12	11	9

Figure3N

2	7	4
5	4	5
6	3	6
4	3	3
3	7	6
5	3	5
4	4	3
4	5	4
5	4	5
3	5	4
3	4	5
6	5	3
3	4	3
4	5	5
4	4	5
5	6	6
4	5	5
3	3	6
6	4	3
4	4	5
4	4	3
6	3	4
5	4	5
6	4	2
4	2	2

Figure4C

LD	LD+LAC	LL
1.1	1.8	1.8
1	2.1	1.6
0.9	2.1	1.7
0.8	2.2	1.6
0.8	2	1.5

Figure4D

LD	LD+2DG	LL
1.1	0.6	1.6
1	0.7	1.4
0.9	0.5	1.5
0.8	0.4	1.7
0.8	0.6	1.6

Figure4E

LD	LD+LAC	LL
1.1	1.8	2
0.9	2.1	1.7
0.8	3	1.4
0.9	2.5	1.5
0.7	2.2	1.8

Figure4F

LD	LD+2DG	LL
1.1	0.5	2
0.9	0.4	1.7
0.8	0.6	1.5
0.9	0.7	1.5
0.7	0.5	1.5

Figure4G

	LL			LL+LAC			LL+2DG	
1	0.84675	0.69256	1.62451	1.70527	1.87905	0.29321	0.42045	0.51051

Figure4I

LL	LL+LAC	LL+2DG
1.21116	3.11476	0.66765
1.48032	2.85101	0.60778
1.46512	3.05146	0.64814

Figure4J

LL	LL+LAC	LL+2DG
0.16834	0.31921	0.14011
0.16416	0.36702	0.13131
0.20514	0.28493	0.12115

Figure4K

	LL			LL+LAC			LL+2DG	
0.32	0.4	0.35	0.65	0.75	0.68	0.24	0.21	0.24

Figure4M

LL	LL+LAC	LL+2DG
32.967	53.618	42.462
33.209	39.886	31.761
49.415	53.174	42.655
40.555	55.097	32.026
46.862	56.56	32.205
45.035	52.504	39.915
47.587	52.887	41.55
50.875	58.498	36.919
52.241	60.883	31.25
33.209	54.429	38.526

Figure4N

	LD			LD+LAC			LD+2DG	
1	1.31521	0.99022	1.60391	1.79202	2.08723	0.73285	0.80195	0.68377

Figure4P

LD	LD+LAC	LD+2DG
37.241	42.439	37.024
40.242	40.438	38.329
38.134	41.349	34.186
37.153	38.753	37.614
40.176	42.951	35.349
38.653	41.137	36.759
39.456	39.423	35.146
38.786	40.279	34.196
37.134	39.613	37.189
38.256	38.461	35.761

Figure5B

Control	H_2O_2
53.261	60.185
50.482	58.649
49.258	50.184
48.369	56.638
56.248	58.963
47.562	55.186
52.177	54.823
49.158	57.358
49.164	54.148
51.687	59.654

Figure5C

	Control		H_2O_2		
0.0015	0.0008	0.0011	0.002	0.0026	0.0021

Figure5D

	Control			H_2O_2	
0.0037	0.0029	0.0031	0.0013	0.0009	0.0013

Figure5E

	Control			H_2O_2	
0.0079	0.0067	0.0065	0.0029	0.0022	0.003

Figure5F

	Control			H_2O_2	
0.0004	0.0004	0.0004	0.0007	0.0007	0.0008

Figure5H

Control	H_2O_2
2.6481	4.3186
2.8578	4.6152
2.6155	4.5199

Figure5J

LL	LL+DPI
55.621	41.648
40.365	30.552
52.349	38.496
54.185	37.453
55.165	31
48	40.452
52.498	38.453
58.649	43.547
57.449	31.469
52.946	40.863

Figure5K

	LL			LL+DPI	
0.0004	0.0005	0.0004	0.0002	0.0002	0.0003

Figure5M

LD	LL	LL+DPI
0.6048	1.6373	0.5601
0.5123	1.7468	0.5567
0.4216	1.6147	0.5315

Figure6B

LL	LL+MK
0.7439	0.4708
0.7794	0.5698
0.7659	0.5215

Figure6C

LL	LL+MK
0.9683	0.8944
0.9213	0.8855
0.9437	0.8325

Figure6E

LD	LD+DPI	LD+LPS	LD+LPS+DPI
10	6	10	5
10	5	9	5
13	8	7	7
12	10	5	6
12	9	7	4
8	7	7	2
10	10	10	6
12	11	5	5
9	6	6	4
10	8	7	6
10	8	11	4
9	7	6	6
11	8	6	4
12	10	10	5
10	7	8	2
8	12	10	3
10	9	8	6
12	9	10	4
9	10	6	5
12	9	6	6

Figure6G

LL	LL+LAC	LL+LAC+DPI	LL+LAC+MK
8	10	15	10
12	17	16	8
15	12	8	10
8	9	8	12
11	17	9	12
15	12	9	14
9	15	10	9
16	10	10	8
11	13	8	12
10	18	12	10
11	13	13	9
12	14	7	10
11	17	8	11
14	15	15	9
15	13	10	10
11	12	7	11
15	11	11	9
10	18	7	10
12	13	12	
11		8	
11		12	
15		12	
		8	

Figure6I

LL+LPS	LL+LPS+LAC	LL+LPS+LAC+DPI	LL+LPS+LAC+MK
10	12	5	3
12	14	9	9
13	15	5	6
12	12	10	8
10	15	8	9
13	15	7	6
10	13	7	8
11	15	9	10
13	14	6	7
10	17	7	5
13	12	12	8
14	15	6	5
14	13	7	11
13	14	3	3
12	7	7	6
15	16	7	7
12	14	5	6
9	9	9	8
6	8		
5	8		
8	10		
8			

Original image:

related to Figure1 J:

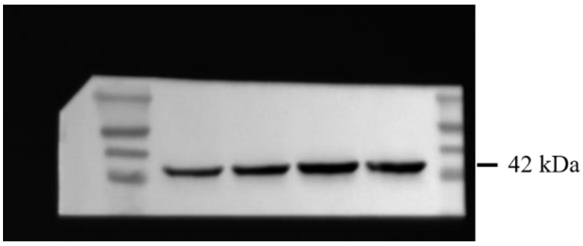

β-actin

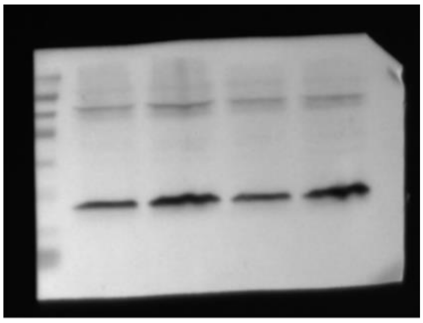

H3K18

related to Figure1 R:

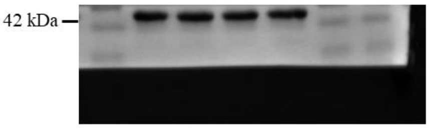

β-actin

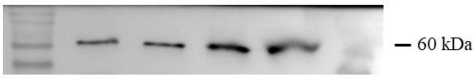

P-Akt

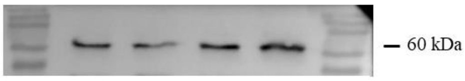

Akt

related to Figure3 A and C:

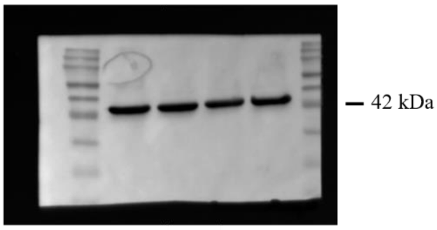

β-actin

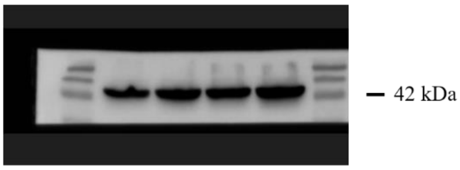

β-actin

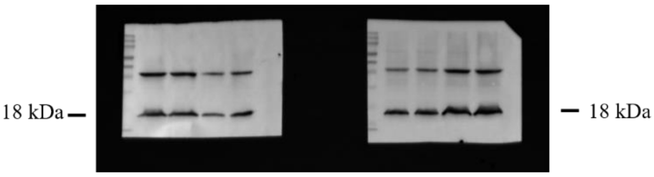

H3K18

related to Figure4 H：

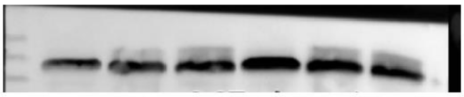

42 kDa

β-actin

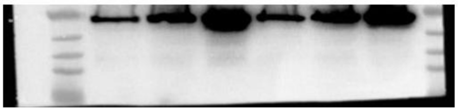

60 kDa

Akt

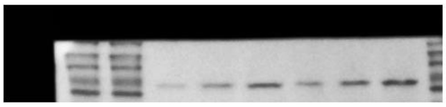

60 kDa

P-Akt

related to Figure5 G and L：

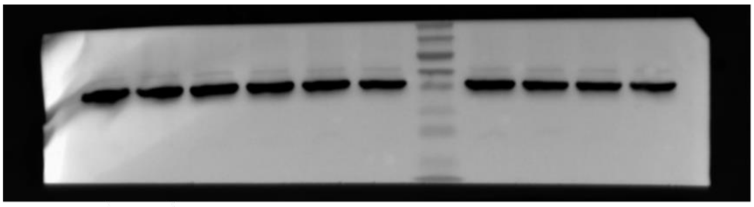

42 kDa

β-actin

18 kDa

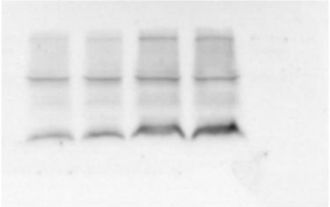

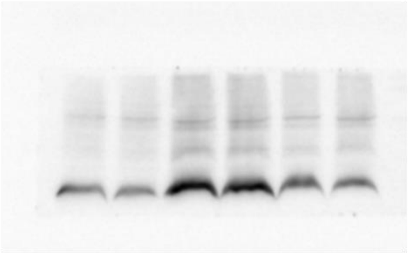

18 kDa

H3K18

H3K18

related to Figure6 A：

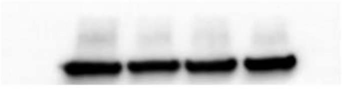

— 42 kDa

β-actin

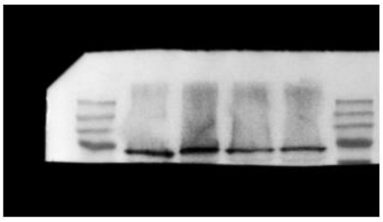

— 60 kDa

Akt

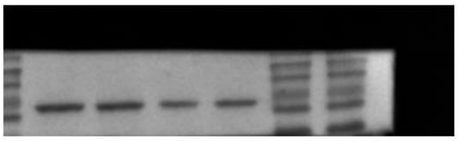

— 60 kI

P-Akt
