# Supplementary material for: Histone lactylation-ROS loop contributes to light exposure-exacerbated neutrophil recruitment in zebrafish
Source: Commun Biol. 2024 Jul 20;7:887. doi: 10.1038/s42003-024-06543-5 (PMC11271584; doi:10.1038/s42003-024-06543-5)
Supplement: Supplementary file 4 — Reporting Summary [file 42003_2024_6543_MOESM4_ESM.pdf]

nature portfolio

Corresponding author(s): da-long ren

Last updated by author(s): 2024-6-9

Reporting Summary

Nature Portfolio wishes to improve the reproducibility of the work that we publish. This form provides structure for consistency and transparency in reporting. For further information on Nature Portfolio policies, see our Editorial Policies and the Editorial Policy Checklist.

Statistics

For all statistical analyses, confirm that the following items are present in the figure legend, table legend, main text, or Methods section.

n/a	Confirmed	
☐	☒	The exact sample size (*n*) for each experimental group/condition, given as a discrete number and unit of measurement
☐	☒	A statement on whether measurements were taken from distinct samples or whether the same sample was measured repeatedly
☐	☒	The statistical test(s) used AND whether they are one- or two-sided<br>*Only common tests should be described solely by name; describe more complex techniques in the Methods section.*
☒	☐	A description of all covariates tested
☐	☒	A description of any assumptions or corrections, such as tests of normality and adjustment for multiple comparisons
☐	☒	A full description of the statistical parameters including central tendency (e.g. means) or other basic estimates (e.g. regression coefficient) AND variation (e.g. standard deviation) or associated estimates of uncertainty (e.g. confidence intervals)
☐	☒	For null hypothesis testing, the test statistic (e.g. *F*, *t*, *r*) with confidence intervals, effect sizes, degrees of freedom and *P* value noted<br>*Give P values as exact values whenever suitable.*
☒	☐	For Bayesian analysis, information on the choice of priors and Markov chain Monte Carlo settings
☒	☐	For hierarchical and complex designs, identification of the appropriate level for tests and full reporting of outcomes
☐	☒	Estimates of effect sizes (e.g. Cohen's *d*, Pearson's *r*), indicating how they were calculated

Our web collection on statistics for biologists contains articles on many of the points above.

Software and code

Policy information about availability of computer code

Data collection	Leica Microscope Software (version X) were used to acquire images.
Data analysis	Quantification of neutrophil was performed by ImageJ (v1.8.0). Statistical analysis was performed byl GraphPad prism 8.4.3.

For manuscripts utilizing custom algorithms or software that are central to the research but not yet described in published literature, software must be made available to editors and reviewers. We strongly encourage code deposition in a community repository (e.g. GitHub). See the Nature Portfolio guidelines for submitting code & software for further information.

Data

Policy information about availability of data

All manuscripts must include a data availability statement. This statement should provide the following information, where applicable:

- Accession codes, unique identifiers, or web links for publicly available datasets
- A description of any restrictions on data availability
- For clinical datasets or third party data, please ensure that the statement adheres to our policy

All data generated during our study are fully documented in the published article and its supplementary information. The source data behind the graphs in the paper are provided in Supplementary Data 1.

Human research participants

Policy information about studies involving human research participants and Sex and Gender in Research.

Reporting on sex and gender	No human research participants were involved.
Population characteristics	No human research participants were involved.
Recruitment	No human research participants were involved.
Ethics oversight	No human research participants were involved.

Note that full information on the approval of the study protocol must also be provided in the manuscript.

Field-specific reporting

Please select the one below that is the best fit for your research. If you are not sure, read the appropriate sections before making your selection.

☒ Life sciences ☐ Behavioural & social sciences ☐ Ecological, evolutionary & environmental sciences

For a reference copy of the document with all sections, see nature.com/documents/nr-reporting-summary-flat.pdf

Life sciences study design

All studies must disclose on these points even when the disclosure is negative.

Sample size	For experiments involving imaging and quantification, numbers of animals were > 20.
Data exclusions	No data were excluded from the analysis.
Replication	Gene expression evaluation and phenotype experiments were repeated at least 3 times, All attempts of replication were successful.
Randomization	Animals used in the experiments were cultured in same environment and selected randomly to control or experimental groups.
Blinding	The investigators were blinded to group allocation during data collection and analysis.

Reporting for specific materials, systems and methods

We require information from authors about some types of materials, experimental systems and methods used in many studies. Here, indicate whether each material, system or method listed is relevant to your study. If you are not sure if a list item applies to your research, read the appropriate section before selecting a response.

Materials & experimental systems

n/a	Involved in the study	
☐	☒	Antibodies
☒	☐	Eukaryotic cell lines
☒	☐	Palaeontology and archaeology
☐	☒	Animals and other organisms
☒	☐	Clinical data
☒	☐	Dual use research of concern

Methods

n/a	Involved in the study	
☒	☐	ChIP-seq
☒	☐	Flow cytometry
☒	☐	MRI-based neuroimaging

Antibodies

Antibodies used	Akt (CST, 4691T) .H3K18la (PTMBIO, PTM-1427RM).
Validation	All antibodies have been validated by company by demonstrating Western blot and Immunocytochemistry (see website).

Animals and other research organisms

Policy information about studies involving animals; ARRIVE guidelines recommended for reporting animal research, and Sex and Gender in Research

Laboratory animals	Zebrafish(WT/AB).Zebrafish(lyz:EGFP).
Wild animals	No wild animals were used in this study.
Reporting on sex	zebrafish embryo/larvae were used in this study.
Field-collected samples	No field-collected samples were used in this studyField-collected samples Ethics oversight.
Ethics oversight	We strictly follow the guidelines and regulations of sYXK (Anhui) 2016-007, Animal Resources Center, Anhui Agricultural University.

Note that full information on the approval of the study protocol must also be provided in the manuscript.
